# Sexual violence among young women in Nigeria: a cross-sectional study of prevalence, reporting and care-seeking behaviours

**DOI:** 10.4314/ahs.v23i1.31

**Published:** 2023-03

**Authors:** Anthony Idowu Ajayi, Christiana Alake Alex-Ojei, Bright Opoku Ahinkorah

**Affiliations:** 1 Sexual, Reproductive, Maternal, New-born, Child and Adolescent Health (SRMNCAH) Unit, African Population and Health Research Centre, APHRC Campus, Manga Close, Nairobi, Kenya; 2 Department of Demography and Social Statistics, Federal University Oye-Ekiti, Nigeria; 3 School of Public Health, Faculty of Health, University of Technology, Sydney, Australia

**Keywords:** Sexual violence, rape, coerced sex, alcohol use, adolescents, young women, Nigeria

## Abstract

**Background:**

Only a few studies, mostly hospital-based, have examined sexual violence among young people women in Nigeria. We examined the prevalence, correlates, perpetrators, reporting of and health-seeking behaviour for sexual violence using data of 395 young women (aged 17-24) obtained from a Nigerian university.

**Methods:**

We selected participants using stratified sampling and defined sexual violence as sexual acts or attempts to obtain sexual acts by violence or coercion by any person irrespective of their relationship to the victim. Descriptive and inferential statistics were used to summarise the data obtained.

**Results:**

Our analysis shows that 39.5% of the respondents had experienced some form of sexual violence. Adequate family financial support was protective against sexual violence after adjusting for all background characteristics (AOR: 0.60 95% CI: 0.39-0.93). However, young women who use alcohol had higher odds of reporting sexual violence experience than those who never used alcohol. In most cases (78.5%), perpetrators were well known to the victims. Only 3.3% of sexual violence cases were reported to law enforcement agencies and just 13.1% of victims sought care from health providers.

**Conclusion:**

Sexual violence occurs at a tragically high frequency, and victims rarely report incidents to law enforcement agencies or access the much-needed care. The findings suggest a need for interventions that address why victims of sexual violence rarely report to law enforcement or seek care.

## Background

Despite receiving significant attention from the government and many developmental partners, sexual violence remains a burdensome and pervasive public health challenge, affecting millions of women and girls globally. Approximately 6% of women globally and in sub-Saharan Africa (SSA) have experienced some non-partner sexual violence at least once in their lifetime^1^. About 27% of women have experienced sexual and/or physical violence from an intimate partner at least once in their lifetime. This translates to 753 million women globally. However, prevalence of sexual and/or physical violence from an intimate partner is higher in SSA, affecting 33% of women^1^. Some sub-Saharan African countries like the Democratic Republic of Congo (47%), Liberia (43%), Uganda (45%), Gabon (41%), South Sudan (41%), Zambia (41%), Burundi (40%), and Lesotho (40%) have the highest prevalence of lifetime physical and/or sexual intimate partner violence among ever-married/partnered women aged 15–49 years globally^1^. It is estimated that 15 million adolescent girls aged 15 to 19 have ever had incidences of forced sex globally 2. Adolescents and young women living in poor neighbourhoods and those who are physically or intellectually disabled are disproportionately exposed to and affected by sexual violence 3–7.

The effects of sexual violence on victims' physical and mental health are damaging and lifelong. Persons who suffered sexual violence are more likely to report high levels of post-traumatic stress disorder, psychological distress, depression, and suicidal ideation 3,4,8–13. Untreated psychological distress exposes victims to untoward suffering, and hampers their ability to function well in their work and enjoy a good quality of life^14^. In some cases, physical injuries from the assault result in hospitalisation and drains victims limited financial resources 4,9,10,12. Also, due to the fear of stigma and reprisal from their assailants, victims of sexual violence rarely report incidence^6^,^12^,^15^ or receive medical treatment, thereby exposing them to a heightened risk of contracting HIV and other sexually transmitted diseases as well as unwanted pregnancy^16^–^18^.

Scholars have argued that sexual violence against women is enshrined in male hegemonic cultures that view women as inferior to men and thus, assume that women's bodies are available for men's sexual exploitation 5, 19. Besides male hegemonic culture, poverty is one of the notable risk factors for sexual violence^20^. The rate of sexual violence is higher among vulnerable and poor women and girls because perpetrators of sexual violence often intentionally target them and they are less likely to report or be believed when they do 20. Women who had economic support from their spouses and those who were financially independent were less likely to suffer sexual violence than those who did not have such support 21.

Also, rape and all forms of sexual abuse and violence are common in the presence of drug and alcohol abuse^4^,^22^. Even though alcohol does not cause sexual violence, it can facilitate it. The relationship between alcohol use and sexual violence is bidirectional.^23^ A study has found that boys use alcohol to intoxicate their girlfriends and lower their resistance to sexual advances before raping them 24. Also, rape is more likely to occur in settings like parties and bars, where alcohol is consumed 22,25. Alcohol use is associated with aggression, loss of inhibition, and increased sexual arousal 26, 27. On the other end, girls and women who were sexually assaulted may use alcohol to cope with the traumatic effect of the incident 23. Moreover, peer pressure has been reported as among the risk factor for rape in South Africa. Boys reported forcing their girlfriends to have sex with them due to peer pressure from their friends 6,19.

Like most countries in SSA, Nigeria has not adequately established a framework for the prosecution of rape as a crime 28. There is also limited knowledge of handling rape victims and collecting evidence to prosecute perpetrators by health workers 28, 29. The main barrier to achieving progress in this regard is the lack of adequate marshalling of evidence in advocacy. While studies on sexual violence are limited 8, 30–33, they suggest, nonetheless, that sexual violence prevalence ranges from 22.2%^8^ to 53.2%^7^. Victims are mostly young females, perpetrators are mainly males, and victims rarely report or seek care 12 ,30 ,32, 34. However, these studies are mostly hospital-based and have not adequately established and characterised the burden of different forms of sexual violence among young persons.

Also, recent data on the burden of sexual violence, reporting, and treatment access among young women are lacking. However, existing studies from different contexts have shown that sexual violence occurs at a higher rate among adolescent girls and young women than any other age cohort 2 ,21 ,35. They also have a higher risk of acquiring HIV, unintended pregnancy, unsafe abortion, and deaths from pregnancy-related causes 16, 36–38. Besides, adolescents have unique developmental needs and violence experienced at this stage can have significant and lifelong impacts in ways unique from those who are first assaulted when they are older. It is important to study sexual violence among adolescents and young adults based on this background. Our study addresses this gap by expanding our knowledge on rape and sexual violence among young persons in Nigeria. We examine the prevalence and predictors of sexual violence and incidence reporting, reasons for not reporting, and health-seeking behaviours of victims. Our findings will inform current debates about sexual violence in the country.

## Methods

We conducted a cross-sectional survey among female students of a university in Southwest Nigeria, between June and November 2019. Students were chosen as the target population for this study because studies have documented high rates of sexual violence on campus and among young people^35^, ^39^. The selected university has a diverse group of students because it is federally owned, and admission is given to students from all parts of the country. Students live in hostels and residences in and around the university campus. Participants were included if they were registered students of the university and aged 17 to 24 years. Visitors were excluded.

### Study population and sampling procedure

A sample size of 400 was needed to determine the prevalence of sexual violence at the confidence level (α) of 95%, 5% margin of error, 7,500 female students, 50% population proportion, and 5% possible attrition. Stratified sampling was used to select the study respondents. Students were stratified by year, and school of study and population proportionate to the size of level and school of the study were selected (Table S1). A graduate student—with prior experience in conducting large surveys—was recruited as a research assistant to administer questionnaires using Open Data Kit (ODK). The research assistant was trained for this study and on ethical considerations guiding the research. We allocated the sample size to the eight schools and five levels of study. The research assistant prioritised each school and level of study and recruited students from their classrooms. For example, the research assistant visited School of Sciences and began with first-year students. Once the sample size allotted to them was achieved, the research assistant moved to second-year students until he completed the survey in the School of Sciences. This was done over six months until the study sample was achieved. Respondents completed the questionnaire on the research assistant's mobile phone or participants' phone in private spaces earmarked for the study. The ODK collect application offers the respondents privacy and allows them to respond to the questions freely. On average, an interview was completed in 25 minutes. We did not provide any monetary reward for participation. Also, no identifying information was collected from participants. Participation was voluntary, and respondents were informed about the purpose of the study, the use of data, how privacy will be ensured, and their right to confidentiality. We provided the phone contact of a clinical psychologist for all respondents to contact for counselling if they experienced distress. The study was granted ethical approval by the review committee of the University of Fort Hare, South Africa (Reference number: GON011) and Ondo State Ministry of Health in Nigeria.

### Variables and measurements

Sexual violence was defined as sexual acts or attempts to obtain sexual acts by violence or coercion by any person irrespective of their relationship to the victim. The main outcome of interest was the lifetime prevalence of sexual violence. We used three questions to examine the experience of sexual violence among respondents, given that using multiple questions has demonstrated more reliable estimates. Respondents responded to three questions, including, 1) Has anyone ever touched your genitals inappropriately without your permission or consent? 2) Has anyone attempted to force you to have sex with him? 3) Has anyone forced you to have sex with him? Responses were classified as “yes” and “no”. We probed respondents to infer if they told anyone, how long ago it happened, who was told, who the perpetrator was, if the incident was reported to the police, if psychological counselling or post rape treatment were received and how the incident has affected them. Open-ended questions were used to explore reasons for not reporting the incidents to law enforcement, health-seeking behaviours, and the impact of the incident on their lives.

### Covariates

Based on previous studies, we included three sets of covariates, including individual, behavioural, and family level covariates. The individual-level covariates included were age, marital status, and orphanhood status. Age was measured as a continuous variable. The behavioural covariates included were drug, alcohol and tobacco use and attending social activities like clubbing and partying. Responses to these variables were categorised as “yes” or “no”.

The family level covariates included were family financial support, family structure, living with parents, and parent-child communication about abstinence and methods of preventing STIs and unintended pregnancy. We specifically asked respondents to rate the level of financial support they receive from their family as adequate, moderate, insufficient and no support. Family financial support was used as a proxy for family wealth in this study, given that students may not know their parents' income. We further asked them to rate the overall and emotional support they receive from home. Family structure was classified as single-parent family, two-parents family, polygamous family, and foster family. Also, we asked respondents to answer with “yes” or “no” on whether they live with each of their parents and if they have discussed abstinence and STI and unintended pregnancy prevention with their parents.

### Data Analysis

We downloaded the data in Stata format from the server and cleaned it to remove outliers. The cleaning involved running frequency counts and percentages to identify outliers or data entry errors. Five respondents with missing responses were removed from the analysis. The five participants dropped out of the study early and did not answer questions on sexual violence. Given that missing cases were few, there was no need for a sensitivity analysis. Frequency, percentage, mean and standard deviation were computed for variables of interest. Also, we used Pearson chi-square to examine the association between sexual violence and individual, family and behavioural level factors. We also fitted adjusted and unadjusted logistic regression models with a 95% confidence interval to examine predictors of sexual violence. Variables were included in the model based on findings of previous similar studies^7^, ^8^ ,^20^ ,^23^ indicating the potential association of the variable with the study outcome. We estimated Pearson correlation to examine if there is multicollinearity among the variables, but the results showed no evidence of collinearity. Alpha values less than 0.05 were deemed to be statistically significant. The qualitative data derived from our two open-ended questions on reasons for not reporting the incidents to law enforcement officers and how it affected them were coded and grouped into themes. Eight themes emerged as reasons for not reporting the incidents to police and these themes were described using narratives, frequency, and percentage counts. Four themes emerged on the impacts of sexual violence on their health and well-being.

## Results

### Descriptive findings

We presented the demographic, behavioural and family characteristics of 395 study participants with complete responses in Table 1. Most participants were aged 20-24 (67.8%), received adequate family support (72.2%), from the nuclear family (70.9%), never used alcohol (73.2%), tobacco (91.9%) and drugs (90.6%). While almost all respondents had received abstinence-only education from their parents, only 6.6% had received contraceptive information from their parents.

**Table 1:** Demographic, behavioural and family characteristics of respondents

Variables	Frequency	Percentage
Age		
17–19	128	32.4
20–24	267	67.6
Relationship status		
Married	9	2.3
Dating and cohabiting	25	6.3
Dating but not cohabiting	146	37.0
Not in a relationship	215	54.4
Family support		
Adequate	285	72.2
Moderate	100	25.3
Insufficient	8	2.0
No support	2	0.5
Financial support		
Adequate	241	61.0
Moderate	136	34.4
Insufficient	15	3.8
No support	3	0.8
Family structure		
Nuclear	280	70.9
Polygamous	42	10.6
Single parent	61	15.4
Foster parent	12	3.0
Father alive		
Yes	344	87.1
No	51	12.9
Live in the same household as father		
Yes	307	77.7
No	22.3	22.3
Mother alive		
Yes	380	96.2
No	15	3.8
Live with mother		
Yes	360	91.1
No	35	8.9
Alcohol use		
Ever used alcohol	106	26.8
Never used	289	73.2
Drug use		
Current user	3	0.8
Previously user	34	8.6
Never used	358	90.6
Cigarette smoking		
Current user	6	1.5
Previously user	26	6.6
Never used	363	91.9
Parental communication on how to prevent STIs and pregnancy		
Yes	26	6.6
No	369	93.4
Parental communication on abstinence		
Yes	387	98.0
No	8	2.0
Attending of social activities like clubbing and partying		
Yes	56	14.2
No	339	85.8

**Figure 1:**
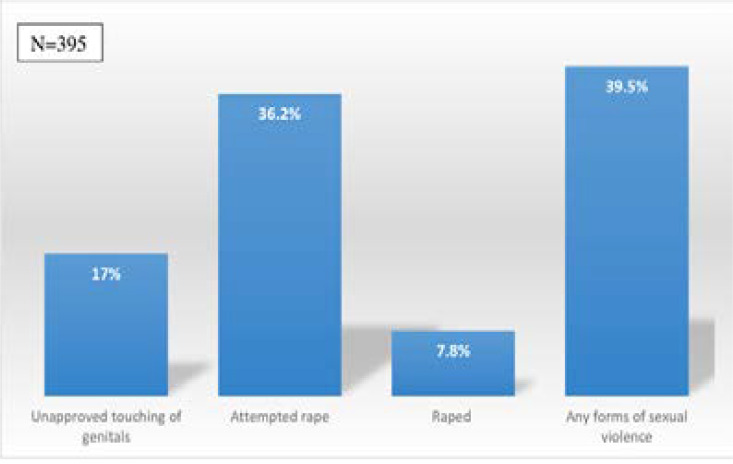
Prevalence of sexual violence

Figure 1 presents the prevalence of sexual violence among respondents. Overall, 39.5% of respondents had experienced some form of sexual violence. While 36.2% reported attempted rape, 7.8% had been raped, and 17% had had their genitals touched without their consent.

As shown in Table 2, one-third of girls aged 17-19 reported having experienced sexual violence at some time in their lives. By age 24, 42% reported lifetime experience of sexual violence. Higher prevalence of sexual violence was reported among girls who received inadequate family support (50.0%) and financial support (47.4%), from polygamous and single-parent family (47.0%), have ever been in a relationship (42.4%), currently use alcohol (58.1%), and do not live with their mothers (54.3%) compared to those who received adequate family support and financial support, those who lived in two-parent households, those who had never been in a relationship, those who had never used alcohol and those who lived with their mothers.

**Table 2:** Sexual violence by background characteristics

Variables	Lifetime experience of sexual violence	Unapproved touching of genitals	Attempted rape	Raped
Age				
17–19	43 (33.6)	18 (13.1)	39 (30.5)	5 (3.9)
20–24	113 (42.3)	49 (18.4)	104 (39.0)	26 (9.7)
P-value	0.060	0.179	0.062	0.030
Relationship status				
Married or dating and cohabiting	12 (35.3)	4 (11.8)	12 (35.3)	3 (8.8)
Dating but not cohabiting	67 (45.9)	27 (18.5)	62 (42.5)	14 (9.6)
Not in a relationship	77 (35.8)	36 (16.7)	69 (32.1)	14 (6.5)
P-value	0.137	0.637	0.131	0.552
Family support				
Adequate	101 (35.4)	38 (13.3)	94 (33.0)	19 (6.7)
Inadequate or Moderate	55 (50.0)	29 (26.4)	49 (44.5)	12 (10.9)
P-value	0.006	0.002	0.022	0.117
Financial support				
Adequate	83 (34.4)	33 (13.7)	74 (30.7)	15 (6.2)
Inadequate or Moderate	73 (47.4)	34 (22.1)	69 (44.8)	16 (10.4)
P-value	0.007	0.022	0.003	0.096
Family structure				
Nuclear	102 (36.4)	39 (13.93)	91 (32.5)	21 (7.5)
Polygamous/ single parent family/ foster family	54 (47.0)	28 (24.3)	52 (45.2)	10 (8.7)
P-value	0.052	0.012[AA1]	0.017	0.688
Orphanhood status				
Both parents alive	130 (38.9)	51 (15.3)	118 (35.3)	29 (8.7)
One or both parents dead	26 (42.6)	16 (26.2)	25 (41.0)	2 (3.3)
P-value	0.342	0.032	0.241	0.112
Live in the same household as father				
Yes	115 (37.5)	44 (14.3)	103 (33.6)	23 (7.5)
No	41 (46.6)	23 (26.1)	40 (45.5)	8 (9.1)
P-value	0.078	0.009	0.028	0.382
Live with mother				
Yes	137 (38.1)	56 (15.6)	126 (35.0)	26 (7.2)
No	19 (54.3)	11 (31.4)	17 (48.6)	5 (14.3)
P-value	0.046	0.021	0.081	0.127
Alcohol use				
Ever used	55 (51.9)	25 (23.6)	50 (47.2)	11 (10.4)
Never used	101 (35.0)	42 (14.5)	93 (32.2)	20 (6.9)
P-value	0.002	0.034	0.006	0.258
Ever been in a relationship				
Yes	139 (42.4)	59 (18.0)	128 (39.0)	30 (9.1)
No	17 (25.4)	8 (11.9)	15 (22.4)	1 (1.5)
P-value	0.006	0.153	0.006	0.019
Social activities engagement				
Yes	29 (51.8)	14 (25.0)	28 (50.0)	7 (12.5)
No	127 (37.5)	53 (15.6)	115 (33.9)	24 (7.1)
P-value	0.042	0.084	0.020	0.162
Parental communication of pregnancy and STI prevention				
Yes	11 (42.3)	4 (15.4)	10 (38.5)	3 (11.5)
No	145 (39.3)	63 (17.7)	133 (36.0)	28 (7.6)
P-value	0.761	0.0825	0.804	0.469

Similarly, the prevalence of unapproved touching was higher among young women who received inadequate family support (26.4%) and financial support (22.1%), did not live with their parents (31.4%), alcohol users (32.3%), and orphans (26.2%) than their counterparts. The prevalence of attempted rape was highest among young women aged 20-24 years (39%), dating but not cohabiting (42.5%), who received inadequate family support (44.8%), alcohol users (51.6%) and who do not live with their mother (48.6%). Lifetime experience of rape was higher among those aged 20-24 (9.7%), and those who have ever dated (9.1%) than those aged 17-19 and those who never dated. These findings are from bivariate analysis and were considered preliminary to guide the multivariable analysis

### Multivariable analysis

Results from the multivariable analysis showed that adequate family financial support was protective against sexual violence after adjusting for all background characteristics (AOR: 0.60 95% CI: 0.39-0.93). Also, young women who use alcohol (AOR: 1.98 95% CI: 1.23-3.19) had higher odds of experiencing sexual violence compared with those who never used alcohol (Table 3).

**Table 3:** Adjusted and unadjusted models showing the predictors of sexual violence

Variables	Unadjusted Odds Ratio	Adjusted Odds Ratio
Age		
17–19	0.69 (0.44–1.07)	0.90 (0.56–1.46)
20–24	1	1
Ever been in a relationship		
Yes	2.16 (1.20–3.91) *	1.74 (0.92–3.28)
No	1	1
Family support		
Adequate	0.55 (0.35–0.86) *	-
Inadequate or Moderate	1	-
Family financial support		
Adequate	0.58 (0.39–0.88) *	0.60 (0.39–0.93) *
Inadequate or Moderate	1	1
Family structure		
Nuclear	0.65 (0.42–1.01)	0.71 (0.36–1.40)
Polygamous/ single parent family/ foster family	1	1
Orphanhood status		
Both parents alive	0.86 (0.49–1.49)	1.57 (0.74–3.34)
One or both parents dead	1	1
Live in the same household as father		
Yes	0.69 (0.43–1.11)	0.92 (0.42–2.03)
No	1	1
Live with mother		
Yes	0.52 (0.26–1.04)	0.51 (0.23–1.12)
No	1	1
Alcohol use		
Current user	2.01 (1.28–3.15) *	1.98 (1.23–3.19) *
Never used	1	1
Attends social activities like clubbing		
Yes	1.79 (1.02–3.17) *	1.45 (0.29–1.20)
No	1	1

There was collinearity between family support and financial support; thus, only one was included in the adjusted model

### Reporting of sexual violence

Of the 156 young women who had experienced sexual violence, 153 responded to further questions on the incidents and were considered in the analysis in this section. About 42 percent of those who had experienced sexual violence (n=153) told someone about the incidence (Table 4). Most of those who informed someone told their friends (51.6%) and parents (34.4%). In most cases (78.5%), perpetrators were well known to the victims, with friends (51.0%) and boyfriends (18.3%) being the main perpetrators of sexual violence. Only 3.3% of cases were reported to law enforcement agencies. Several reasons emerged from the analysis of the reasons for not reporting to the law enforcement agencies, including “fear”, “shame”, “young and naïve”, “self-blame”, “parent(s) resolved it”, “lack of trust in the police”, “protection of privacy”, “it was a failed attempt”, “got an apology”, and “it was someone close”. Overall, most survivors of sexual violence (54.9%) did not report the incidence to law enforcement agencies because they considered it unnecessary given it was only an attempted rape, and the perpetrator failed. Fear of being shamed also played a considerable role in not reporting incidents to law enforcement, as alluded to by one-tenth of the victims. Survivors affirmed that society is judgmental and often shames victims. As such, the involvement of law enforcement officers could expose them to public scrutiny, shame, and ridicule. One survivor described this as “a second rape”. To protect their privacy, they often avoid reporting to law enforcement officers. Lack of trust of law enforcement officers and lack of evidence also played a role in victims' decision not to report the incident.

**Table 4:** Reporting of sexual violence, care seeking and effect of incidence

Variables	Frequency N=153	Percentage
Tell someone		
Yes	64	41.8
No	89	58.2
Who was told		
Friend	33	51.6
Parents	22	34.4
Siblings/pastor	9	14.0
Perpetrator		
Friend	78	51.0
Boyfriend	28	18.3
Uncle/brother	11	7.1
Stranger	33	21.5
Other people such as a cousin, employer, neighbour, lecturer, and family friend	8	5.2
Made report to law enforcement		
Yes	5	3.3
No	148	96.7
Reasons for not reporting		
It is just an attempt, did not succeed	84	54.9
Afraid	15	9.8
Ashamed	12	7.8
Parent resolved it/ I got an apology/ Boyfriend fought him	12	7.3
Young and naïve/ I do not know how to go about it/self-blame	5	3.4
It is my boyfriend/ It's a close relative	8	5.3
I do not trust the police/ I do not know him/ lack of evidence	7	4.7
I want to keep it private	10	6.5
How the incidence has impact survivors		
Depressed	28	18.3
Shocked/traumatised	19	12.5
Emotionally destabilised, hatred of men and isolation/ ashamed/ embarrassed	27	17.6
Not affected, it was an attempt	79	51.6
Received and form of care, including from a clinical psychologist		
Yes	20	13.1
No	133	86.9

Slightly over half of the victims affirmed that the incident did not affect them at all, and they further explained that this was so because it was only an attempt and the perpetrators failed. Depression, trauma, shock, and shame were the commonly used words to describe the aftereffect of the incidence. Only one in ten victims of sexual violence received any form of care, including from a clinical psychologist.

## Discussion

We examined the prevalence and predictors of sexual violence, reporting, reasons for not reporting, self-reported effects of the incidents, and victims' care-seeking behaviour, using a sample of young women in a tertiary institution in Nigeria. We found that about two-fifths (39.5%%) of young women had experienced some form of sexual violence, with most of them reporting attempted rape.

Regarding the prevalence of sexual violence, our findings corroborate the findings of previous studies 13 and particularly those conducted among students in tertiary institutions in Nigeria 7 ,8 ,32. Our prevalence of 39.5% is higher than the 22.2% reported by Iliyasu et al.^8^ but lower than the 46.7% reported in Mezie-Okoye and Alamina 32 and 53.2% found in Umana, Fawole and Adeoye 7. The differences in prevalence can be attributed to variations in sexual violence definitions, differences in the study setting and population, and the study period. Nevertheless, the high prevalence of sexual violence among young women in Nigeria could be attributed to the pervasive male hegemonic culture^5^ ,^19^. As found in this study and consistent with previous studies 30 ,34, 40, an overwhelming majority of cases are not reported to law enforcement, indicating that justice was not served and heightening the probability of perpetrators' repeating the offense. Without reporting and punishing the assailants, it will be very difficult to end sexual violence. Thus, it is important to understand and address the main reasons victims rarely report cases.

In this study, slightly over half of the victims did not report the incidence to law enforcement agencies because they consider an unsuccessful rape attempt not to warrant such a drastic step. Extant literature has reported similar findings, highlighting that unacknowledged victims are less likely to report incidents to law enforcement or disclose it to anyone^39^, ^41^. When survivors can identify what happened to them as a crime, they are often more likely to report it. Others were too afraid or ashamed, young and naïve, preferred to protect their privacy, and did not trust the police. Previous studies have indicated that victims usually lack confidence in the ability of security agencies to bring the perpetrators to justice and because they are afraid of the stigma attached to being identified as a victim of rape or sexual assault 10,15. This is more likely to occur in situations when the perpetrator is a close family member or intimate partner 12 ,33 ,42. We found in this study that parents sometimes settle and resolve cases of sexual violence without involving law enforcement. Often the rights and interests of victims are not adequately protected as apology only is accepted as sufficient retribution. As such, settling cases of sexual violence by parents without legal representations should be discouraged. Consistent with previous studies 28, 29, our findings also show that a few victims fail to go through the justice system due to barriers such as inadequate evidence, and ignorance regarding the legislative framework governing the treatment of sexual violence cases on the part of victims. Also, perpetrators and actors in the justice system, shortage of medical professionals to stand in judicial proceedings as expert witnesses, and the attachment of different meanings to sexual violence by different social groups, contrary to legal definitions, are other reasons discouraging victims from reporting to law enforcement in previous studies 28 ,29. While it is important to encourage victims to report incidents, reforming the systems and agencies is even more critical to ensure that victims are not met with blame, shame, disbelief, and other negative responses when they decide to report.

More concerning, however, is the lack of care and support for victims of sexual violence despite many reporting experiences of depression, anxiety, post-traumatic stress, shock, self-blaming, and shame. This is consistent with previous studies, some of which reported additional experiences such as sexual dysfunction, somatic complaints, sleep disturbances, withdrawal from relationships, and attempted suicide as other consequences of sexual violence 4, 9, 11 ,13. Our study shows that only one in ten victims received any medical help, including from a psychologist. Lack of medical care, especially in cases of rape, could expose victims to unwanted pregnancy, HIV, and STIs 16–18 ,36. Also, untreated psychological distress resulting from the experience of rape or attempted rape could harm victims' immediate and long-term mental health, affecting their ability to enjoy affectionate relationships and hindering their quality of life. Investing in judgment-free care and support for victims of sexual violence is critically needed and would yield remarkable returns in terms of good health and enhanced ability to contribute to the economy.

Lastly, we found that alcohol use increases the odds of exposure to sexual violence and adequate family support is protective against sexual violence. The association between alcohol use and sexual violence is well documented^23^–^27^. While alcohol does not cause sexual violence, it could enhance its occurrence, including being used as leverage to inebriate women and girls 24. Also, previous studies have reported the link between poverty and sexual violence 20 ,43. Older and wealthy men could use money to leverage a relationship with young girls, thus exposing them to sexual violence. In part, girls who received adequate family financial support were less likely to follow men to bars and clubs in the hope of receiving gifts or money 44. Therefore, dealing with the risk factors for sexual violence requires interventions that empower girls financially and discourage the use of alcohol.

## Strengths and limitations

The major strength of this study was the use of both open and close-ended questions to examine the phenomenon of sexual violence among young women in the most populous country in Africa. The focus on young women is another strength of the study as these constitute the next generation of women whose current reproductive health challenges can affect the future reproductive health of the country. The sample size used for the study and the use of a standard sample size determination technique to obtain the sample size also bolsters the validity of the findings. Notwithstanding, given that sexual violence was self-reported, there is a possibility of social desirability bias in this study resulting in under-reporting of cases of sexual violence. However, privacy was ensured via the Open Data Kit app, and multiple questions were used to mitigate the under-reporting of sexual violence.

Our findings on the association between alcohol use and exposure to sexual violence should be interpreted with caution, given that we only measured the lifetime use of alcohol and drugs and did not ask respondents if they used these substances immediately before the assault. Lastly, our sample of young women is generally more educated than the population of this cohort in the country, thus limiting the generalisability of the findings. However, our results are similar to a population-based study among young people in a section of the country. Ultimately, more nationally representative studies are needed to better inform our understanding of sexual violence among young people in the country.

## Policy and public health implications

This study has both policy and public health implications. In terms of policy, our findings emphasised the need to strengthen existing sexual violence prevention policy and intervention strategies in Nigeria. First, there is a need to increase girls' level of education on sexual violence and their level of confidence in care and justice-seeking. Second, there is a need for community-based engagement to combat discrimination against sexual violence victims. Third, the community and family also have roles in educating boys about responsible sexual behaviours, the importance of consent, and the right to bodily autonomy. Lastly, there is a need to strengthen health systems to provide medical and psychological support for victims and laws and enforcement to ensure stiff penalties for perpetrators.

## Conclusion

Sexual violence not only occurs at a tragically high frequency, victims also rarely report incidents to the police and do not access much-needed care after incidences of sexual violence. There is a need to design and implement interventions that address the reasons victims rarely report or access care. Such intervention should address victim-blaming and shaming and train law enforcement officers on responsible, judgment-free, and professional codes of ethics dealing with victims. Effective strategies of linking victims of sexual violence to medical care should be developed and implemented in Nigeria to address the huge gaps in access to care among victims of sexual violence.

## Data Availability

The data analysed will be made available by the corresponding author on reasonable request.

## Abbreviations

ODK: Open Data Kit
HIV: Human Immunodeficiency Virus
STI: Sexually Transmitted Infection
